# Morphological and molecular evidence support the taxonomic separation of the medically important Neotropical spiders *Phoneutria depilata* (Strand, 1909) and *P. boliviensis* (F.O. Pickard-Cambridge, 1897) (Araneae, Ctenidae)

**DOI:** 10.3897/zookeys.1022.60571

**Published:** 2021-03-08

**Authors:** Nicolas A. Hazzi, Gustavo Hormiga

**Affiliations:** 1 The George Washington University, Department of Biological Sciences, Washington, D.C. 20052, USA The George Washington University Washington, D.C United States of America; 2 Fundación Ecotonos, Cra 72 No. 13ª-56, Cali, Colombia Fundación Ecotonos Cali Colombia

**Keywords:** Andes, Maxent, niche conservatism, Phylogenetics, species delimitation

## Abstract

The species of the genus *Phoneutria* (Ctenidae), also called banana spiders, are considered amongst the most venomous spiders in the world. In this study we revalidate *P. depilata* (Strand, 1909), which had been synonymized with *P. boliviensisis* (F.O. Pickard-Cambridge, 1897), using morphological and nucleotide sequence data (COI and ITS-2) together with species delimitation methods. We synonymized *Ctenus peregrinoides*, Strand, 1910 and *Phoneutria colombiana* Schmidt, 1956 *with P. depilata*. Furthermore, we designated *Ctenus signativenter* Strand, 1910 as a *nomen dubium* because the exact identity of this species cannot be ascertained with immature specimens, but we note that the type locality suggests that the *C. signativenter* syntypes belong to *P. depilata*. We also provide species distribution models for both species of *Phoneutria* and test hypotheses of niche conservatism under an allopatric speciation model. Our phylogenetic analyses support the monophyly of the genus *Phoneutria* and recover *P. boliviensis* and *P. depilata* as sister species, although with low nodal support. In addition, the tree-based species delimitation methods also supported the separate identities of these two species. *Phoneutria boliviensis* and *P. depilata* present allopatric distributions separated by the Andean mountain system. Species distribution models indicate lowland tropical rain forest ecosystems as the most suitable habitat for these two *Phoneutria* species. In addition, we demonstrate the value of citizen science platforms like iNaturalist in improving species distribution knowledge based on occurrence records. *Phoneutria depilata* and *P. boliviensis* present niche conservatism following the expected neutral model of allopatric speciation. The compiled occurrence records and distribution maps for these two species, together with the morphological diagnosis of both species, will help to identify risk areas of accidental bites and assist health professionals to determine the identity of the species involved in bites, especially for *P. depilata*.

## Introduction

The species of the genus *Phoneutria* are considered aggressive and amongst the most venomous spiders in the world (Foelix 2010). Currently this genus includes eight large (17–48 mm) nocturnal species that are widely distributed in Central America and South America (Simó and Brescovit 2001; Martins and Bertani 2007). Their venom has a neurotoxic action and many researchers have analyzed its components and the epidemiology of the bites of these species (Gomez et al. 2002; Richardson et al. 2006; Bucaretchi et al. 2008, 2018; Garcia et al. 2008). Most of the clinically relevant bites by this genus are caused by *P. nigriventer* (Keyserling, 1891) and occur in Brazil (around 4,000 cases per year), with only 0.5% being severe (Bucaretchi et al. 2018).

*Phoneutria boliviensis* (Pickard-Cambridge, 1897) is a widespread species distributed from Central America (Costa Rica) to central South America (Bolivia), found across many types of ecosystems and geographical barriers that commonly divide other taxa (e.g. the Andes mountain system that separates many cis and trans Andean lowland lineages) (Bartoleti et al. 2018; Salgado-Roa et al. 2018). This species was originally described from the “Madre de Dios” Amazonian region in Bolivia and only the male palp was illustrated because the epigynum of the single female was damaged (Schiapelli et al. 1973). Schiapelli et al. (1973) illustrated the epigynum and the male palp based on other specimens identified by F.O. Pickard-Cambridge as *P. boliviensis* (a female from Ecuador and a male from Charaplaga, Bolivia). These authors report that male specimen at The Natural History Museum (at their time known as the British Museum of Natural History) in the vial with the syntypes of *Ctenus boliviensis* was a specimen in good condition of *P. nigriventer* (Keyserling, 1891). Subsequently, Simó and Brescovit (2001) indicated that they were not able to find the type specimens, and therefore they considered them lost.

At that time, *P. boliviensis* was known only to occur in the Amazon region, until Valerio (1983) reported this species in Costa Rica. Later, Simó and Brescovit (2001) revised the genus and synonymized several ctenids with *P. boliviensis*. Simó and Brescovit (2001) acknowledged the large morphological variation of *P. boliviensis* across its distribution range, but they interpreted this variation as intraspecific and diagnosed it by the truncated apex of the male retrolateral tibial apophysis.

During field work in Costa Rica, Panama, Colombia, Ecuador and Peru, and careful examination of museum specimens from these countries, we realize that *P. boliviensis* can be separated into two distinct species. One trans-Andean species, *Phoneutria depilata* and the true *P. boliviensis* (cis-Andean) endemic of the Amazon region. Therefore, in this study we revalidated *P. depilata* that was synonymized with *P. boliviensisis* by Simó and Brescovit (2001) and we designate a neotype of *P. boliviensis* collected from the Madre de Dios region of Peru. We follow an integrative taxonomic approach using molecular, morphological, and ecological data to support the separation of these two species. We also provided species distribution models (SDMs) for both species of *Phoneutria*. Furthermore, we also tested the hypothesis of niche conservatism under an allopatric speciation model (Wiens 2004; Wiens et al. 2011). This hypothesis states that the tendency of lineages to maintain their ancestral ecological niche, and their failure to colonize and adapt to new environments, separate ancestral taxa promoting speciation (Wiens 2004). Therefore, we expect that *P. depilata* and *P. boliviensis*, separated by the Andean mountain, present niche conservatism. *Phoneutria depilata* has been deeply studied in the literature as *P. boliviensis*, in works regarding its venom composition and toxicity (Estrada-Gomez et al. 2015; Valenzuela-Rojas et al. 2019), natural history (Hazzi 2014; Valenzuela-Rojas et al. 2020), geographic distribution (Valerio 1983; Hazzi et al. 2013), bite accidents to humans (Trejos et al. 1971; Florez et al. 2003) and introductions to Europe through banana shipments (Cathrine and Longhorn 2017; Rozwałka et al. 2017). Unlike *P. depilata*, except for brief field anecdotal mentions (Torres-Sánchez and Gasnier 2010), there is no such information for *P. boliviensis*. We have also provided additional information on the natural history of both species.

## Methods

### Museum abbreviations

The material examined and/or collected belongs to the following museums:

**ICN-AR**Instituto de Ciencias Naturales-Universidad Nacional de Colombia, Bogota (E. Flórez);

**MCZ**Museum of Comparative Zoology, Harvard University, Cambridge, USA (G. Giribet and L. Liebensberger);

**MPUJ**Museo Pontificia Universidad Javeriana, Bogota (D. Forero);

**MUSENUV**Museo Entomológico de la Universidad del Valle, Cali, Colombia (J. Cabra);

**MUSM-ENT** Museo de Historia Natural, Lima, Peru (D. Silva);

**MZUCR**Museo de Zoología, Escuela de Biología, Universidad de Costa Rica (G. Barrantes);

**USNM**National Museum of Natural History, Smithsonian Institution, Washington DC, USA (H. Wood);

**ZMB**Museum für Naturkunde der Humboldt Universität, Berlin, Germany (J. Dunlop).

### Morphological examination and description of species

Specimens were preserved in 95% ethanol. Descriptions and terminology follows Simó and Brescovit (2001) and Martins and Bertani (2007). All measurements were taken in millimeters using the application of LAS in a Leica M205A stereomicroscope. Epigyna were digested with pancreatin solution (Álvarez-Padilla and Hormiga 2007) to enable study of internal structures. Digital images were taken with a Leica DFC425 camera on a Leica M205A stereomicroscope. Extended focal range images were composed using the software package Helicon Focus (version 6.7.1; www.heliconsoft.com) from Helicon Soft Ltd. The SEM images were taken using a LEO 1430VP scanning electron microscope at the Department of Biology of The George Washington University. For scanning electron microscope preparation, structures were cleaned ultrasonically, transferred to 95% and then to 100% ethanol for 10 min in each immersion before being critically-point-dried. The following abbreviations are used: **C** = conductor, **CD** = copulatory duct, **E** = embolus, **ELA** = epigynal lateral apophysis, **ELF** = epigynal lateral field, **ELG** = epigynal lateral guide, **EMF** = epigynal middle field, **FD** = fertilization duct, **IB** = internal bulge of the embolus, **LP** = lateral projection, **MA** = median apophysis, **RTA** = retrolateral tibial apophysis; **S** = spermatheca, **S** = subtegulum.

### DNA-based analysis

*Sampling design.* Due to the widespread climatic niche of *P. depilata*, we sequenced seven specimens from Costa Rica, Ecuador and Panama that were collected from mountain to lowland areas, and from dry to rain forests ecosystems (Table 1). For *P. boliviensis*, we sequenced six specimens collected in three localities distributed from the north through to the southern part of the Peruvian Amazon, including one specimen from the type locality. In addition, we sequenced a specimen of *Phoneutria fera* Perty, 1833 collected in the Amazon of Ecuador and added two more sequences of the same species from GenBank (HM575999 and KY017637). As an outgroup, we sequenced one specimen of *Spinoctenus escalerete*Hazzi et al., 2018, *Ctenus datus* Strand, 1909, C. aff. amphora Mello-Leitão, 1930 and *Kiekie curvipes* (Keyserling, 1881). In addition, we also added a sequence of *Ctenus crulsi* Mello-Leitão, 1930, from GenBank (KY017633.1).

**Table 1.** DNA taxon sampling generated in this study. Letters in the haplotype/allele column indicate if individuals have the same sequence in the COI or ITS-2 markers respectively; * indicate that the sequence is unique.

Species	Code	Country	Locality	Latitude / longitude	COI	ITS-2	Haplotype/allele	Museum code
*Kiekie curvipes*	GH2776	Costa Rica	Tirimbina Reserve	10.4164, -84.1199	MW598451	MW599260	*/*	MCZ IZ 162190
Ctenus aff. amphora	GH2779	Brazil	Roraima	2.7375, -62.075		MW599262	-/*	MCZ IZ 162193
*Ctenus datus*	GH2778	Panama	Gamboa	9.1216, -79.7034	MW598452	MW599261	*/*	MCZ IZ 162191
*Spinoctenus escalerete*	GH2777	Costa Rica	Las Cruces Biological Station	8.7845, -82.9597	MW598442	MW599254	*/*	MCZ IZ 162192
*Phoneutria fera*	GH2794	Ecuador	Liana Lodge	-1.056, -77.524	MW598443	MW599255	*/A	MCZ IZ 162189
*Phoneutria depilata*	GH2793	Ecuador	Caimito, Esmeraldas	0.7005, -80.0741	MW598444	MW599256	*/A	MCZ IZ 162184-1
*Phoneutria depilata*	GH2787	Panama	Gamboa	9.1216, -79.7034	MW598448	MW599256	*/A	MCZ IZ 162179-1
*Phoneutria depilata*	GH2792	Costa Rica	Tirimbina Reserve	10.4164, -84.1199	MW598445	MW599256	*/A	MCZ IZ 162182-1
*Phoneutria depilata*	GH2791	Costa Rica	Cirenas	9.7199, -85.2119	MW598446		*/-	MCZ IZ 162181-1
*Phoneutria depilata*	GH2790	Panama	Puerto Amuelles	8.2841, -82.8691	MW598447		C/-	MCZ IZ 162180-1
*Phoneutria depilata*	GH2789	Costa Rica	San Isidro	10.0182, -84.0551	MW598447	MW599256	C/A	MCZ IZ 162183-1
*Phoneutria depilata*	GH2788	Panama	Puerto Amuelles	8.2841, -82.8691	MW598447	MW599256	C/A	MCZ IZ 162180-2
*Phoneutria boliviensis*	GH2780	Peru	ACP Panguana	-9.6137, -74.9352	MW598450	MW599258	B/C	MCZ IZ 162188-1
*Phoneutria boliviensis*	GH2781	Peru	Reserva Nacional Allpahuayo Mishana, Biological Station “José Alvarez Alonso”	-3.9663, -73.4368	MW598450	MW599259	B/B	MCZ IZ 162185-1
*Phoneutria boliviensis*	GH2782	Peru	Reserva Nacional Allpahuayo Mishana, Biological Station “José Alvarez Alonso”	-3.9663, -73.4368	MW598450	MW599259	B/B	MCZ IZ 162185-2
*Phoneutria boliviensis*	HG2783	Peru	Madre de Dios, Finca Las Piedras	-12.2259, -69.1142	MW598450	MW599258	B/C	MUSM-ENT 54118
*Phoneutria boliviensis*	HG2784	Peru	ACP Panguana	-9.6137, -74.9352	MW598450	MW599258	B/C	MCZ IZ 162188-2
*Phoneutria boliviensis*	HG2785	Peru	ACP Panguana	-9.6137, -74.9352	MW598449	MW599259	A/B	MCZ IZ 162188-3
*Phoneutria boliviensis*	GH2786	Peru	Reserva Nacional Allpahuayo Mishana, Biological Station “José Alvarez Alonso”	-3.9663, -73.4368	MW598449	MW599257	A/*	MCZ IZ 162185-3

Specimens preserved in 95% ethanol were used for DNA extraction using the Qiagen DNEasy kit. Coxae and femora were used for extractions and the rest of the specimen was preserved as a voucher. Two gene fragments frequently used for species recognition and delimitation in spiders (e.g., Montes de Oca et al. 2016; Ballesteros and Hormiga 2018; Salgado-Roa et al. 2018) were amplified for analysis: the mitochondrial cytochrome c oxidase subunit I (~650 bp, COI) and the nuclear internal transcriber subunit 2 (~300 bp, ITS2). The former was amplified using the primers LCO1490 and HCOout (Folmer et al. 1994; Carpenter and Wheeler 1999) and ITS2 was amplified with the primers FITS and RITS (White et al. 1990; Agnarsson 2010) using the conditions previously reported in Ballesteros and Hormiga (2018). Amplified products were sent to Macrogen USA (Rockville, Maryland) for sequencing. Contigs were formed using GENEIOUS 6.0.6 (http://www.geneious.com; Kearse et al. 2012) and COI sequences were checked for stop codon position, then queried against NCBI BLAST nucleotide database to check for contamination. Multiple sequence alignments were completed using the Q-INS-I search strategy using MAFFT. Gaps were treated as missing data for the phylogenetic analysis.

The best partitioning scheme and substitution models were explored using PartitionFinder 2.1.1 using the ‘‘greedy” search strategy and the correction of the Akaike information criterion (AICc). Four partition schemes were used as input data: first, second and third codon position for COI, and ITS-2 as a whole. Phylogenetic analyses were performed using parsimony (MP), maximum likelihood (ML) and Bayesian inferences (BI). The parsimony analyses were carried out in TNT v. 1.5 (Goloboff et al. 2008; Goloboff and Catalano 2016) using 100 random addition sequences followed by TBR branch swapping algorithm and retaining 10 trees per replicate. Branch support was assessed using 1000 replicates of jackknife resampling (Farris et al. 1996). The Bayesian analyses were performed in MrBayes 3.2.6 (Ronquist and Huelsenbeck 2003) running 20 million generations from four Markov Chain Monte Carlo chains (MCMC). Trees and parameters were sampled every 1000 generations, 25% of the trees were discarded as burn-in and the remainder were used to calculate posterior probabilities. To check that the run was long enough for the chains to converge, the probabilities of the marginal parameters were observed in Tracer v. 1.5 (Rambaut et al. 2014b). The maximum likelihood analyses were performed with the package IQ-TREE 1.4.2 (Nguyen et al. 2015) and ultrafast bootstrap (UFBoot) were used as support measure (Minh et al. 2013).

To measure relationships between haplotypes, we constructed haplotype median-joining networks for each marker using PopArt v1.7 (Leigh and Bryant 2015). Due to the small genetic variation found in the allele network of the nDNA, we only calculated genetic distances for the mDNA. Uncorrected genetic distances (uncorrected p-distance) were calculated within and among *Phoneutria* species pairs using MEGA v.10 (Kumar et al. 2018). We performed both genetic distance and tree-based species delimitation methods in order to distinguish species of *Phoneutria*. The Automatic Barcoding Gap Discovery (ABGD) method (Puillandre et al. 2012) was used to identify breaks between the intraspecific and interspecific diversity (this is known as the barcode gap). This method relies on just pairwise genetic distances and therefore does not used phylogenetic information. Because ABGD was designed for single locus analysis, we only used this method with the COI sequences data. The analysis was performed through the web-server (https://bioinfo.mnhn.fr/abi/public/abgd/abgdweb.html) using default settings and the uncorrected p-distances option.

The three remaining methods used are tree-based. First, we applied the general mixed Yule coalescent model (GMYC, Pons et al. 2006; Fujisawa and Barraclough 2013) using GMYC web server (https://species.h-its.org/gmyc/). This method models the Yule and coalescent processes on an ultrametric tree to determine the transition between intra and interspecific divergences. The ultrametric tree was estimated in BEAST 2.6.0 (Bouckaert et al. 2014) using a coalescent constant population as a tree prior. An uncorrelated relaxed clock with log normal distribution and GTR+Gamma substitution model for each codon was applied. We ran the analysis with 20 million generations of MCMC. Trees and parameters were sampled every 1000 generations, 25% of the trees were discarded as burn-in and the remainder were used to calculate posterior probabilities. To check that the run was long enough for the chains to converge, the probabilities of the marginal parameters were observed in Tracer v.1.5 (Rambaut et al. 2014b). TreeAnnotator version 2.6.0 (BEAST package) was used to build maximum clade credibility trees. For the second method, we applied a Bayesian framework of the multi-rate Poisson tree process (mPTP, Kapli et al. 2017). This approach differs from GMYC in modelling coalescent and speciation events as relative to numbers of substitutions rather than time (Kapli et al. 2017). The minimum branch length was calculated and used as an input together with a likelihood tree (estimated as above). We ran the alignment with 2 independent replicates of MCMC of 5,000,000 generations, sampling every 1000 with a burn-in of 10% of the total length of the chain. GMYC and mPTP were designed to model single locus data, and because ITS-2 market lumped the three morphologically diagnosable species in one, we only show the results with COI.

Finally, we applied the Bayesian Phylogenetics and Phylogeography software (BPP, Yang 2015), a is species delimitation approach based on the Multi Species Coalescent Model. This method uses a Bayesian modelling framework to estimate posterior probabilities of species assignment’s multilocus gene trees, considering uncertainties in the coalescent process. We carried out joint species delimitation and species tree estimation (A11 analysis), assigning individuals *a priori* to a species based on the phylogeny and morphology. For the root age of the tree (*τ*) and the ancestral population size (*θ*), four combinations of priors were used. Combinations were among deep divergence times (*τ* = *G* (1, 10)) and shallow divergence times (*τ* = *G* (2, 2000)), and large populations sizes (*θ* = *G* (1, 10)) and small populations sizes (*θ* = *G* (2, 2000)). We performed 100,000 iterations, sampling every 2, using the 10% of the chain as burn-in. Because mDNA has a different mutation rate and effective population size than nDNA, we did analysis with mDNA+nDNA and mDNA alone. As mDNA obtained similar results, we only provide the results of the multilocus dataset. Currently, all species delimitation methods differentiate simplifying assumptions on the potential real parameter space relevant to species delimitation. Therefore, any of these assumptions could be violated easily in a particular empirical system, consequently only congruently delimited lineages across the different methods were considered as species (Carstens et al. 2013).

### Species model distributions and niche comparisons

**Figure 1.**
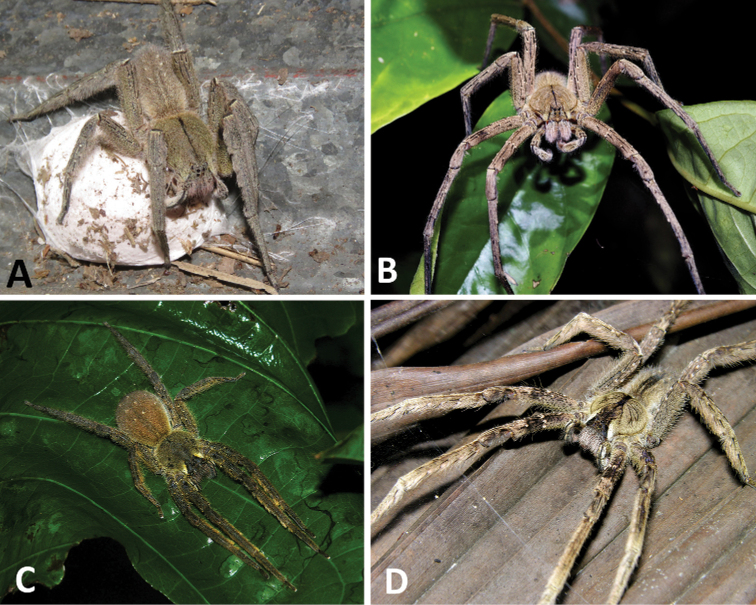
**A–D** Habitus of *Phoneutria* spp. **A** female of *P. depilata* with eggs sac (from Chiriquí, Panama) **B** female of *P. depilata* (from Barro Colorado Island, Panama) **C** female of *P. boliviensis* (from Madre de Dios, Peru) **D** male of *P. boliviensis* (from Napo, Ecuador).

We estimated the distribution of *P. boliviensis* and *P. depilata* using the Maxent algorithm (Phillips et al. 2006; Elith et al. 2011). We used occurrence records from the literature, fieldwork and museum specimens examined by us (herein after LIFIMU database). In addition, we used iNaturalist (https://www.inaturalist.org/) as a novel procedure in spiders to obtain more distribution records for these species. iNaturalist is a citizen science platform that provides unprecedented access to documenting species diversity and distribution across the world (Hochmair et al. 2020). Users upload media (mostly images) of biological findings to the iNaturalist data portal that are later identified to some taxonomic level by the iNaturalist community. Because in most cases, spiders can only reliably be identified by examining their genitalia under the stereoscope, these new apps that rely on images for species identification have not been used on spiders, to our knowledge. However, for these medically relevant spiders, it is possible to identify them using only images (Fig. 1A–D). In the case of *P. depilata*, after extensive fieldwork and the study of museum specimens, we have been able to conclude that this is the only *Phoneutria* species distributed in the Trans Andean region reaching Central America (Nicaragua). Thus, we can assign with high certainty *Phoneutria* images from these regions to *P. depilata*. *Phoneutria boliviensis* is endemic to the Amazonian region and it co-occurs with two more species of *Phoneutria*: *P. fera* and *P. reidyi* (F. O. Pickard-Cambridge, 1897). However, *P. boliviensis* is the only species that has two conspicuous lateral white bands in the anterior area of the carapace (Fig. 1C, D, 4A). In addition, males of *P. boliviensis* have dark black grooves in the carapace (Fig. 1D, 4A). Therefore, *Phoneutria* images from the Amazon region with these coloration features were identified as *P. boliviensis*.

To mitigate the impact of uneven sampling in our occurrence data, we applied a distance correction by taking only one point within a radius of 10 km. We obtained 19 bioclimatic predictor layers summarizing annual trends, seasonality and extremes in precipitation and temperature at a spatial resolution of 30 arc-seconds (i.e. 1 km^2^) from the WorldClim database (Fick and Hijmans 2017). In order to reduce collinearity of the predictor variables, we selected the following variables (Pearson <0.7): annual mean temperature (Bio1), mean diurnal range (Bio2), temperature seasonality (Bio4), annual precipitation (Bio12), precipitation seasonality (Bio15) and precipitation of warmest quarter (Bio18). The modelling area selected for *P. depilata* was the trans-Andean region until Nicaragua and for *P. boliviensis* the Amazon and Orinoquia basins (cis-Andean region). We selected these regions considering species accessible area M (diagram by Barve et al. 2011) based on the geographical extension of gathered records of both species and the distribution of terrestrial ecoregions(Olson et al. 2001) and biogeographic regions of endemism (Morrone 2014) in the Neotropical Region.

We ran the models selecting a logistic output and random seed, and the maximum number of background points maintained at 10,000. To assess model performance, we applied k-fold cross validation procedure splitting the occurrences into training and testing records (70% and 30%, respectively), and replicating this process 15 times. Models were evaluated using the Area Under the Curve Metric (AUC) that compares model results with null expectations using a threshold-independent measure. We average the AUC values obtained in the replicates and created confidence intervals values to assess model significance from random model expectations (AUC > 0.5). In order to make the binary distribution maps, habitat suitability values were converted in presence and absence using the 5^th^ percentile as the threshold value (Liu et al. 2005, 2013). In addition, areas with high probability of presence, but disjunct from areas where specimens have been recorded, were excluded from the prediction (Helgen et al. 2013).

To test niche conservatism among these two species, we used the niche similarity test (Peterson et al. 1999) and niche equivalency test (Graham et al. 2004) in the R package *Ecospat* (Di Cola et al. 2017). First, we performed an environmental principal component analysis (PCA-env) (Broennimann et al. 2012), calibrated with the accessible areas of the two species. We then created a grid of 100 × 100 cells over the ordination space, and a kernel density function was applied on the occurrence data in order to estimate Schoener’s D index (Schoener 1968) with the first to principal components. This metric estimates niche overlap and D values ranging from zero, when niches do not overlap, and one, when niches completely overlap. Finally, the niche equivalence test and the niche similarity test were performed using 1000 simulated replicates in the R package *Ecospat* (Di Cola et al. 2017). Both metrics assess the statistical significance of a measured niche similarity against null model niches taken randomly from the modelling area. However, while niche equivalency test is estimated comparing the empirical D value with random relocation of the occurrence records on different distribution ranges (species lineages), the similarity test is estimated through random shifts of the niches within the available conditions of the study area (Warren et al. 2008; Broennimann et al. 2012).

## Results

### Phylogenetic and species delimitation analyses

**Figure 2.**
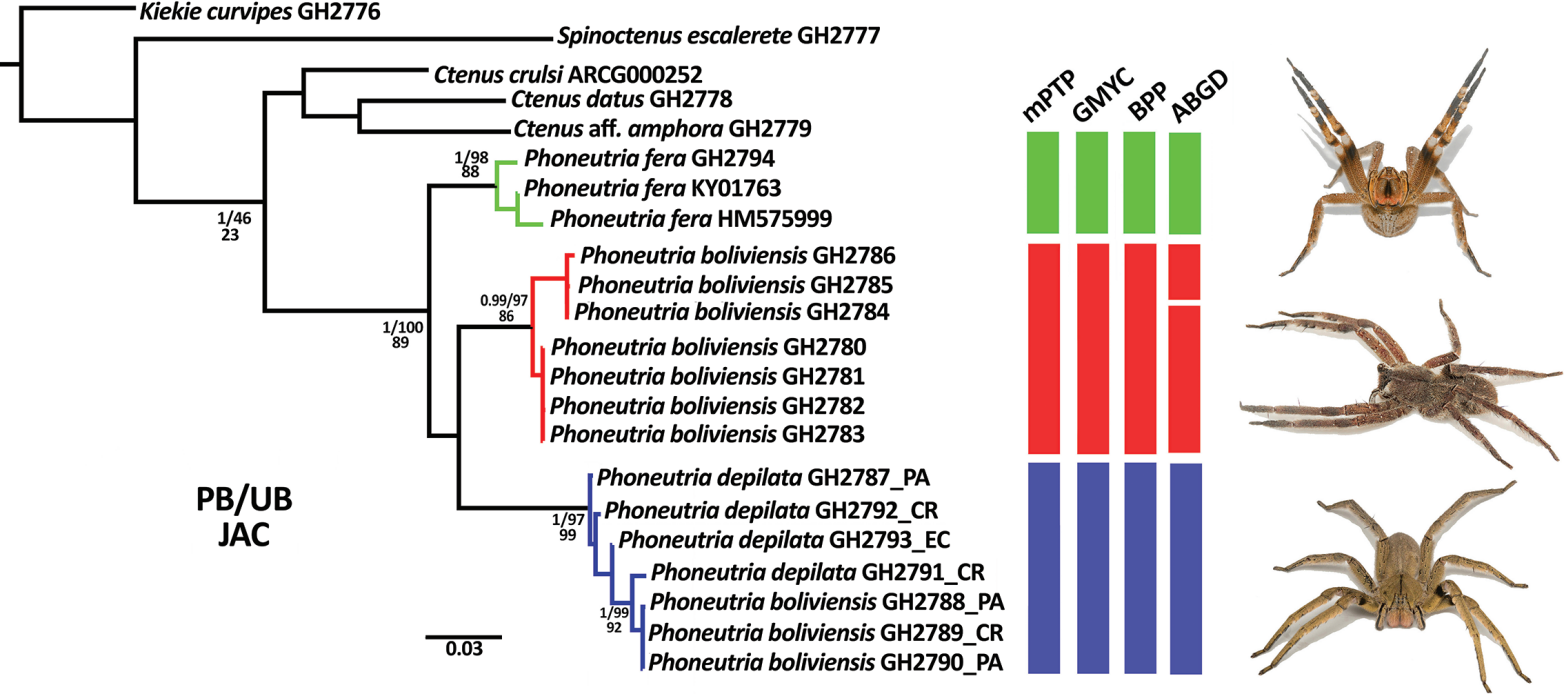
Maximum likelihood phylogenetic tree of the concatenated alignment of COI and ITS-2 markers. PB = posterior probabilities (derived from the Bayesian tree), UB = ultrafast bootstrap (derived from the likelihood tree), and JAC = jackknife (derived from the parsimony tree). Support metrics for nodes with low support (UB and PB < 0.95, and Jac < 70) are not shown. *Phoneutria* images: *P. fera* (top), *P. boliviensis* (center), *P. depilata* (bottom).

The tree topologies of the parsimony, Bayesian and maximum likelihood analyses were congruent in recovering with high support metrics the monophyly of the genus *Phoneutria* and the three morphologically recognized species: *Phoneutria depilata*, *P. fera* and *P. boliviensis*. Therefore, only the likelihood tree is shown (Fig. 2), and the main discrepancies amongst analyses relate to the relationships of *Phoneutria* species mentioned below. While the likelihood and parsimony analyses indicated that *P. boliviensis* is the sister species of *P. depilata*, the Bayesian analysis suggests that *P. boliviensis* is the sister species of *P. fera*. The incongruent nodes receive very low support values of jackknife, posterior probabilities, and ultrafast bootstraps.

**Figure 3.**
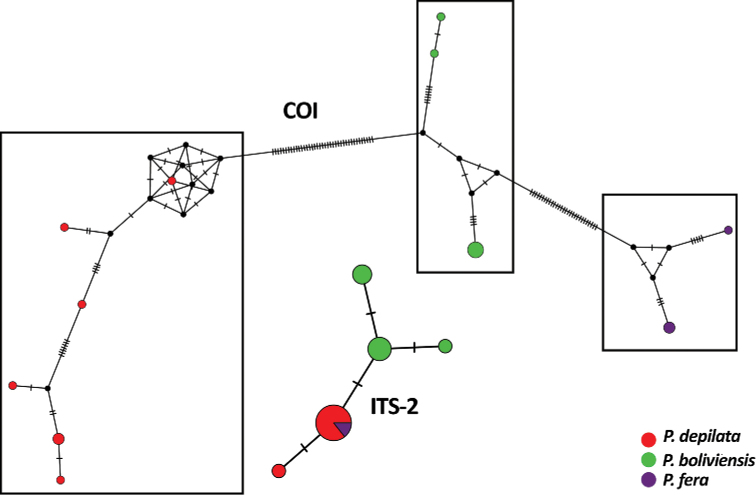
Median joining haplotype network of the COI and ITS-2 markers. Each tick mark on the network branches represents a mutation step and the three black boxes indicate the three haplogroups.

mtDNA haplotype networks (Fig. 3) revealed three major haplogroups that were congruent with the three species clades found in the phylogenetic analyses. *Phoneutria fera* haplotypes were separated from *P. boliviensis* by 29 mutations, and *P. boliviensis* was separated from *P. depilata* by 39 mutations. However, nDNA network (Fig. 3) shows that *P. depilata* and *P. fera* share alleles, and alleles of *P. boliviensis* are separated from this group just by one mutation (Fig. 3). Average genetic mDNA distance for *P. depilata*-*P. fera* was 8.2%, *P. depilata*-*P. boliviensis* 7.4%, and *P. fera*-*P. boliviensis* 6.1%. For intraspecific variation comparisons, the mean p-distance for *P. depilata* was 2%, *P. fera* 1% and *P. boliviensis* 1%.

**Figure 4.**
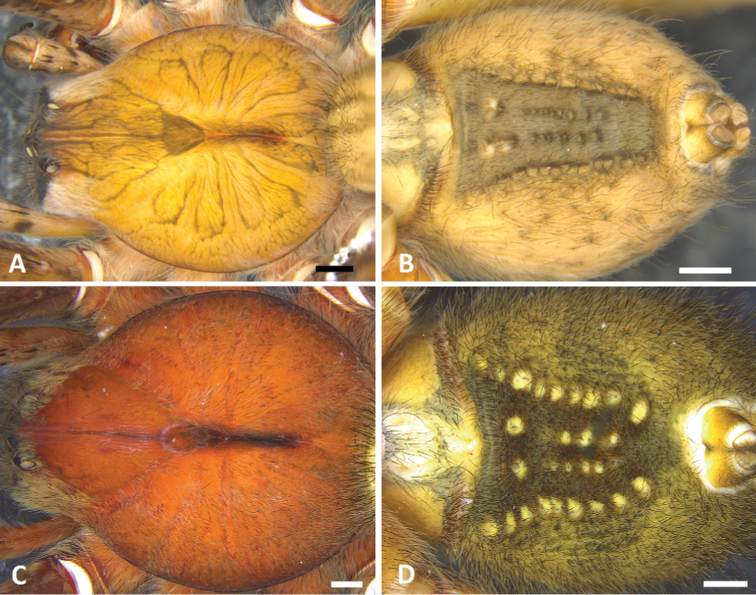
**A, B** dorsal view of the carapace and ventral view of the abdomen of *P. boliviensis* (male from from Finca Las Piedras, Madre de Dios, Peru) **C, D** dorsal view of the carapace and ventral view of the abdomen of *P. depilata* (male from Chiriquí, Panama). Scale bars: 2.00 mm.

The ABGD method indicates four species, separating two specimens of *P. boliviensis* (GH2782 and GH2783) as a separate species. Instead, the mPTP species delimitation analysis indicated with high support (ASV = 0.99) the delimitation of three morphologically recognizable species. In addition, The GMYC analysis produces the same result. The posterior probabilities for the three species in each model tested in BPP were always many times higher than the alternatives scenarios: 0.97 for deep divergence and large population size, 0.33 for deep divergence and small population size (the next most likely scenario was one species with 0.10), 0.71 for shallow divergence and small population size (the next most likely scenario was two species with 0.14) and 1.00 for shallow divergence and large population size. Thus, three parameter combinations suggest the same number of species.

### Occurrence records, potential distribution and niche conservatism

The Fig. 11A, B show the compilation of occurrence records for the two species of *Phoneutria* obtained from LIFIMU database and iNaturalist. Qualitatively, iNaturalist records match relatively well with the known distribution range of both species of *Phoneutria*, and the localities where the records came from are the same localities or the same regions of the localities of LIFIMU database. However, it is important to highlight that iNaturalist provided more occurrence records for both species than LIFIMU database. For instance, in *P. depilata* iNaturalist extends its distribution range to Honduras and there is more density of records in the inter-Andean Valley of Magdalena in Colombia, and the Choco region in Ecuador. In the case of *P. boliviensis*, iNaturalist provides more distribution records in the Amazon of Ecuador, where LIFIMU database has only one record.

Distribution models of *P. boliviensis* and *P. depilata* presented high performance compared to random expectations (AUC = 0.84 ± 0.10 SD for *P. boliviensis* and AUC = 0.84 ± 0.06 SD for *P. depilata*). The distribution model of *P. depilata* highlighted areas with different levels of suitability across Central and South America (Fig. 12A), with highest suitability values located in lowland and premontane areas, and from dry to tropical rain forest ecosystems. This species is well distributed in the inter-Andean Valleys of Magdalena and Cauca in Colombia. In addition, *P. depilata* is distributed in many areas of the Choco region of Ecuador and Colombia, and the Caribbean region reaching to Honduras. For *Phoneutria boliviensis*, the distribution model (Fig. 12B) indicated suitable values in lowland ecosystems of the West Amazon including Brazil, Bolivia, Colombia, Ecuador, Peru and small portion of Venezuela (although without a confirmed occurrence record). The Fig. 13A, B depicts the binary maps of the predicted distribution range of both species of *Phoneutria*.

In the niche comparison analysis, the 1^st^ and 2^nd^ axis of the PCA‐env explained 53.49% and 14.19% of the variance, respectively (Fig. 14B). Niche overlap among *P. depilata* and *P. boliviensis* was moderate (D = 0.31, Fig. 14A). In addition, *P. depilata* presented a larger climatic niche area than *P. boliviensis*. The niche equivalency test indicated that climatic niche of these two species are more equivalent than expected by chance (Fig. 14C). Similarity tests also reject the null expectation between the two species (although the p-value of the similarity test *boliviensis*-*depilata* was marginal) (Fig. 14C). Thus, there is more significant climatic niche conservatism than expected by a null models between the two species of *Phoneutria*.

### Taxonomy

#### Family Ctenidae Keyserling, 1877

##### Phoneutria

Taxon classificationAnimaliaAraneaeCtenidae

Perty, 1833

E967FF4F-B34B-5EE3-BE3B-F7E26859EDE1

###### Type species.

*Phoneutria fera* Perty, 1833.

##### Phoneutria boliviensis

Taxon classificationAnimaliaAraneaeCtenidae

(F. O. Pickard-Cambridge, 1897)

75AE4976-01C5-5301-9B20-17C8CBEFC593

**Figure 5.**
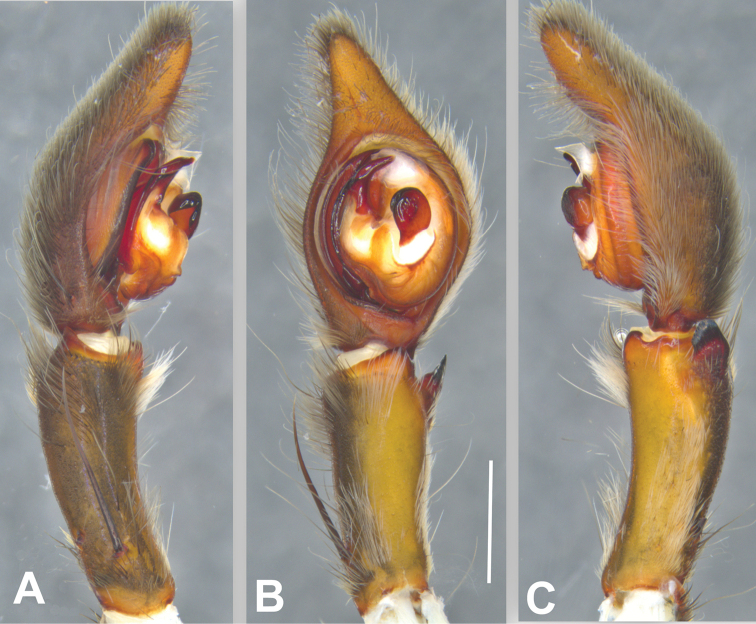
*Phoneutria boliviensis* (from Finca Las Piedras, Madre de Dios, Peru), left male palp **A** prolateral view **B** ventral view **C** retrolateral view. Scale bar: 2.00 mm.

**Figure 6.**
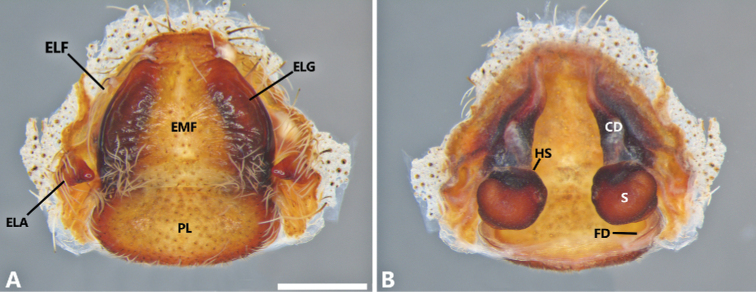
*Phoneutria boliviensis* (Finca Las Piedras, Madre de Dios, Peru), female genitalia **A** epigynum, ventral view **B** vulva, dorsal view. CD = copulatory duct, ELA = epigynal lateral apophysis, ELF = epigynal lateral field, ELG = epigynal lateral guide, EMF = epigynal middle field, FD = fertilization duct, HS = head of spermatheca, PL = posterior lobe, S = spermatheca. Scale bar: 1.00 mm.

Figs 1C, D , 4A, B , 5A, C , 6A, B , 9A, D , 10A, B

Ctenus boliviensis : F. O. Pickard-Cambridge, 1897: 80, pl. 3, (female holotype from Madre de Dios, Bolivia, fig. 3a-c (male), The Natural History Museum, London not found; see Schiapelli and Gerschman de Pikelin 1973: 36, and Simó and Brescovit 2001: 74.
Ctenus nigriventroides Strand, 1907: 426 (female holotype from Bolivia, Museum für Natur und Umwelt der Hansestadt, Lübeck presumed lost; see Eickstedt 1979: 111, and Simó and Brescovit 2001: 74).
Ctenus valdehirsutulus Strand, 1910: 318 (syntypes: female from Sara, W. Bolivia, 60 m, 14 March 1907, J. Steinbach leg., in ZMB 30615; female from Sara, Dpto. Sta. Cruz de la Sierra, Bolivia, 500 m, Steinbach, in ZMB 30616, see Simó and Brescovit 2001: 74).
Ctenus nigriventoides : Petrunkevitch, 1911: 475 (only citation of Strand 1907), 735.
Ctenus chilesicus Strand, 1915: 128 (female holotype from Chile, 1902, O. Hohenemser leg., in SMF-4557).
Phoneutria boliviensis : Schmidt, 1954: 414; 1956: 28; Bücherl 1968: 188; 1969a: 49; Schiapelli and Gerschman de Pikelin 1973: 31, 33–38 (redescription male and female).
Phoneutria nigriventroides : Bonnet, 1958: 3621 (in part, only material from Bolivia); Eickstedt 1979: 111.

###### Neotype

**(herein designated; see comments below). Peru**: Male from Madre de Dios, Puerto Maldonado, Finca Las Piedras (12.2259°S, 69.1142°W, 260 m), 20.IX.2019, N. Hazzi coll. (MUSM-ENT 54118).

###### Justification of the neotype designation.

We have designated a neotype for *P. boliviensis* in accordance with Article 75 of the International Code of Zoological Nomenclature (ICZN 1999). The type material of *Ctenus boliviensis* was considered lost after examination of the spider material at the Natural History Museum, London (Simó and Brescovit 2001). The epigynum of the syntype female was reported to be damaged by Schiapelli and Gerschman (1973). In absence of type material, we consider necessary to designate a neotype to clarify the taxonomic status of *P. boliviensis*. Although the original type locality of *P. boliviensis* is the Madre de Dios area of Bolivia (F.O. Pickard-Cambridge 1897), the locality of the neotype (in Peru) belongs to the same region. The region takes its name from the Madre de Dios river, which is part of the Amazon river watershed. The Madre de Dios basin spreads across Bolivia and Peru. This area is called Inambari and is considered as a single biogeographic area because of its unique composition of species (Da Silva et al. 2005). In addition, the neotype locality is very close to the Bolivian border (30 km in linear distance).

###### Comments.

The syntypes of *Ctenus valderhirsutulus* were revised by Simó and Brescovit (2001) and this species was deemed to be a junior synonym of *Phoneutria bolivienesis*. The syntype localities of *valderhirsutulus* (the Sara Province of Bolivia, in the Santa Cruz Department) are within the distribution area of *bolivienesis* which corroborates the synonymy proposed by Simó and Brescovit (2001). The type of *Ctenus chilesicus* comes from an undisclosed locality in Chile and was deemed to be conspecific with *Phoneutria bolivienesis* by Simó and Brescovit (2001). The records of *Phoneutria* from Chile are of introductions of *P. fera* (Zapfe 1963; Canals et al. 2004). Although we have no reason to question the synonymy of *chilesicus* with *Phoneutria bolivienesis*, which was based on the examination of type specimens, future work should revise the type of *chilesicus*. We suspect that the only specimen of *chilesicus* is an introduction of an already described species (as suggested by Simó & Brescovit) or the result of labeling error.

###### Other material examined.

**Colombia**: Caqueta: two males, Universidad de la Amazonia (1.4998°N, 75.6632°W, 240 m) Florencia, 30.VII.2019, N. Hazzi, L. Martínez, and E. Across-Valencia (MUSENUV); Amazonas: Comunidad Monifue Amena (4.1128°N, 69.9311°W, 70 m) 03.X.2005 (MPUJ). **Peru**: Loreto: two males and two females, Reserva Nacional Allpahuayo Mishana, Biological Station “José Alvarez Alonso” (3.9663°S, 73.4368°W, 120 m) Iquitos, 02.IX.2019, N. Hazzi, E. Vargas and G. Gagliardi (MCZ IZ 162185); one female and one male, Universidad Nacional de la Amazonia Peruana (3.8466°S, 73.3671°W, 110 m), Puerto Almendras, Iquitos, 01.IX.2019, N. Hazzi and E. Vargas (MCZ IZ 162186?); one female, San Rafael (3.5617°S, 73.1191°W, 90 m), 04.IX.2019, N. Hazzi and E. Vargas (MCZ IZ 162187?); Inahuaya, Cerros Orullana (7.1158°S, 75.2709°W, 150 m), 9.VII.1988, R, Fernandez and P. Hocking (MUSM-ENT 511187); Ucayali: four females and four males, Panguana Biological Research Station (9.6137°S, 74.9352°W, 220 m), 15.IV.2019, N. Hazzi (MCZ IZ 162188); Madre de Dios: one female, same data as neotype (MUSM-ENT 054122); one female Zona Reservada de Manu (11.96°S, 71.30°W, 250 m), 01.X.1987, D. Silva & J. Coddington (USNM); three females and one male, Zona Reservada Tambopata (12.83°S, 69.283°W, 290 m) (MUSM-ENT 507653, 507657, 507658 and 507659); Zona Reservada Pakitza (11.96°S, 71.30°W), 26.V.1987, (MUSM-ENT 509196), one male, Explorers Inn (12.8455°S, 69.2942°W), 19.VI.2009 (MUSM-ENT 500807); Santuario Nacional Pampas del Heath (12.042°S, 71.7248°W), 27.VI.1987, V. Morales (MUSM-ENT 509147); Huánuco: one female and one male, Dantas la Molina (9.633°S, 75°W, 270 m), SW Puerto Inca, 18.V.1987 (MUSM-ENT 507582, 511349); San Martin: one female, Juanji (7.1669°S, 76.7395°W, 350 m), 16.VIII.1998 (MUSM-ENT 511348); Pasco: one male, Santa Maria, Rio Palcazu (9.9369°S, 75.2471°W), 8.III.1998, P. Hocking (MUSM-ENT 511043); Amazonas: one male, Condorcanqui (4.59841°S, 77.8599°W), 18.VII.1994, M. Ortega (MUSM-ENT 509062).

###### Diagnosis.

Males of *P. boliviensis* resemble those of *P. depilata* by the truncated apex of the RTA (Fig. 9C, D), but differ by the smaller tegulum (Figs 5B, 9A), round median apophysis enlarged at the base (Figs 5B, 9A), locking lobes located posteriorly (Figs 5B, 9A), in contrast with the narrow base of the median apophysis and pronounced lateral locking lobes in *P. depilata*; and embolus without internal bulge (Figs 5B, 9A). Females of *P. boliviensis* also resemble those of *P. depilata* by the general configuration of the epigynum but differ by the wider area of the EMF (Figs 6A, 10A), copulatory ducts strongly sclerotized (Figs 6B, 10B), and reduced spermatheca heads (Figs 6B, 10B), in contrast with the less sclerotized copulatory ducts and larger spermatheca heads of *P. depilata*. In addition, both females and males can be distinguished from *P. depilata* and the remaining Amazonian species (*P. perty* and *P. fera*) by the two lateral conspicuous white-yellow bands in the anterior area of the carapace which are also absent in all other congeneric species (Fig. 4A).

###### Description.

**Male** (from Madre de Dios, Puerto Maldonado, Finca Las Piedras, Peru; MUSM-ENT 54118). Coloration (Figs 1D, 4A, B): Carapace brown with a longitudinal black line, transversal black stripes and two lateral conspicuous white-yellow bands in the anterior area. Ocular area with dark black-blue setae and back oblique band from PLE to anterior dorsal shield of prosoma edge. Chelicerae brown. Sternum, endites and labium yellowish-brown. Dorsal abdomen yellow-brown, with a longitudinal black line reaching to the median region; ventrally dark brown with four series of pale brown dots. Total length 20.93. Carapace 10.91 long and 13.18 wide, eye diameters: AME 0.41, ALE 0.23, PME 0.72, PLE 0.46. Clypeal height 0.26, sternum 4.57 long, 4.00 wide; labium 1.31 long, 0.84 wide. Leg measurements: I: femur 12.20, patella 4.20, tibia 13.52, metatarsus 17.98, tarsus 5.00, total 52.90 ; II: 17.60, 7.49, 18.81, 13.65, 3.94, total 61.49; III, 14.09, 6.62, 12.42, 8.18, 2.43, total 43.74; IV 11.79, 4.42, 10.8, 12.58, 3.42, total 43.01. Leg spination: I tibia v2-2-2-2-2, d1-1-1, p0-1-0, r1-1-0, metatarsus v2-2-2, p1-0-0 r1-0-0, II tibia v-2-2-2-2-2, d1-1-1, p1-1-0, r1-1-0, metatarsus v2-2-2, p1-0-0 r1-0-0, III v2-2-2, d1-1-1, p1-0-1-0, r1-0-1-0, metatarsus v2-2-2-2, p1-1-2, r1-1-2, IV tibia v2-2-2, d1-1-1, p1-0-1-0, r1-0-1-0, metatarsus v2-2-2-2, d0-1-0, p1-1-2, r1-1-2. Palp. RTA small and truncated at the apex (Figs 5C, 9C); embolus curve without internal bulge (Figs 5B, 9A); cup-shaped median apophysis constrained at the base (Figs 5B, 9A); conductor membranous, hyaline and C-shaped (Figs 5B, 9A); tegulum with probasal rounded projection (Figs 5B, 9A).

**Female** (from Madre de Dios, Puerto Maldonado, Finca Las Piedras, Peru; MUSM-ENT 054122). Coloration (Figs 1C, 4A, B): Carapace brown with a longitudinal black line and two lateral conspicuous white-yellow bands in the anterior area. Ocular area with dark brown setae and back oblique band from PLE to anterior dorsal shield of prosoma edge. Chelicerae brown with red setae. Sternum, endites and labium yellowish-brown. Dorsal abdomen yellow-brown, with a yellow dot; ventrally dark brown with four series of pale brown dots. Total length 20.19. Carapace 9.70 long and 7.57 wide, eye diameter: AME 0.45, ALE 0.29, PME 0.46, PLE 0.53. Clypeal height 0.44, sternum long 3.94 and 3.55 wide, endites 3.89 long and 2.50 wide, labium 1.43 long and 1.25 wide. Leg measurements: I: femur 9.06, patella 3.98, tibia 9.93, metatarsus 8.01, tarsus 2.33, total 33.31; II, 8.45, 4.19, 8.67, 6.90, 2.27, total 30.48; III 6.92, 3.20, 5.97, 5.46, 1.59, total 23.14; IV 8.66, 3.51, 8.06, 9.00, 1.58, total 30.81. Leg spination: tibia I–II v2-2-2-2-2, metatarsus I–II v2-2-2-2-2; III tibia v2-2-2, d1-1-1, p1-0-1-0, r1-0-1-0; metatarsus v2-2-2-2, p1-1-2, r1-1-2; IV tibia v2-2-2, d1-1-1, p1-0-1-0, r1-0-1-0, metatarsus v2-2-2-2, d0-1-0, p1-1-2, r1-1-2. Epigynum (Figs 6A, 9A): middle field convex with straight edges, anteriorly divergent and posteriorly convergent; lateral field with lateral apophysis. Vulva (Figs 6B, 9B): copulatory ducts strongly sclerotized and reduced spermatheca heads, fertilization ducts small and posteriorly located.

###### Variation.

Males (n = 6): Total length 9.70–10.60, carapace 4.86–5.90, femur I 5.90–6.72. Females (n = 5): Total length 12.22–15.22, carapace 6.33–6.97, femur I 5.20–5.86.

###### Distribution.

Lowland tropical rain forests of the Amazon (0–1000 m) in Bolivia, Brazil, Colombia, Ecuador and Peru (Figs 11–13).

###### Natural history.

*Phoneutria boliviensis* is the smallest species of the genus and it inhabits in sympatry with *P. fera* and *P. reidyi*. Torres-Sánchez and Gasnier (2010) indicated that *P. boliviensis* seems to be restricted to periodically indudated forests because they have never been detected in “terra firme” forests. In Peru, this species was also very common in swamp forests (aguajales) dominated by the large, dioecious palm *Mauritia flexuosa*. However, we also found that *boliviensis* is not exclusive to inundated forests but also can be found in “terra firme” forests and even in the Amazonian foothills in Caqueta, Colombia. In these non-inundated ecosystems, *P. boliviensis* is found in secondary forests and forest edges. This species lives in the leaf litter and low vegetation. It is interesting to highlight that in the Amazon of Colombia, Ecuador and Peru, we always found *P. boliviensis* in sympatry with *P. fera* but never with *P. reidyi*.

##### Phoneutria depilata

Taxon classificationAnimaliaAraneaeCtenidae

(Strand, 1909) sp. reval.

7D466905-93B1-58CC-B065-79F83CF10620

**Figure 7.**
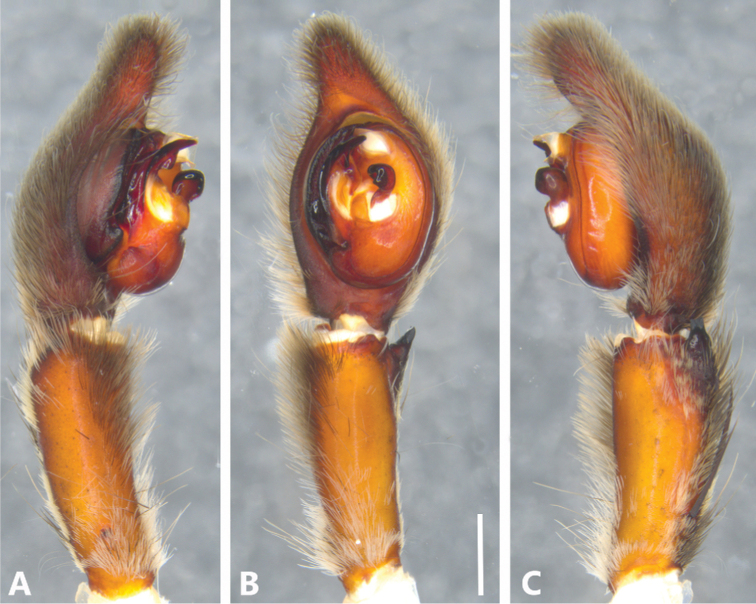
*Phoneutria depilata* (from Puerto Amuelles, Chiriqui, Panama), left male palp **A** prolateral view **B** ventral view **C** retrolateral view. Scale bar: 2.00 mm.

**Figure 8.**
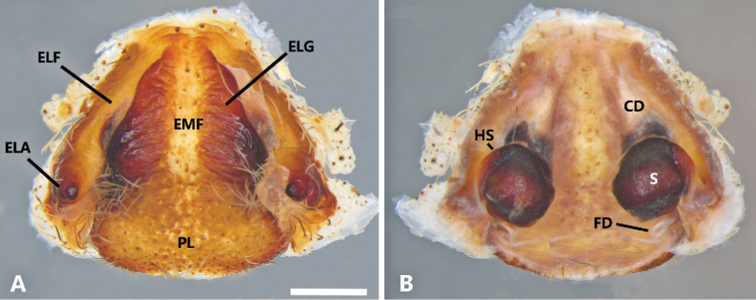
*Phoneutria depilata* (from Puerto Amuelles, Chiriquí, Panama), female genitalia **A** epigynum, ventral view **B** vulva, dorsal view. CD = copulatory duct, ELA = epigynal lateral apophysis, ELF = epigynal lateral field, ELG = epigynal lateral guide, EMF = epigynal middle field, FD = fertilization duct, HS = head of spermatheca, PL = posterior lobe, S = spermatheca. Scale bars: 1.00 mm.

**Figure 9.**
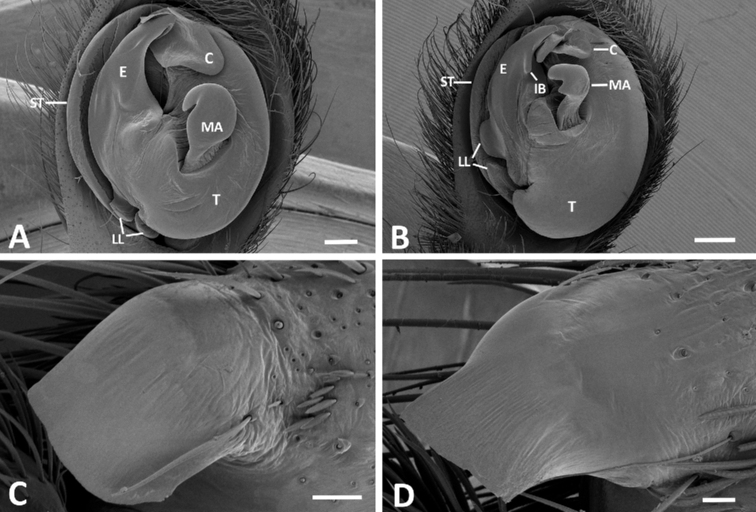
**A, B** ventral view of the male palp of *P. boliviensis* (from Pucallpa, Peru) and *P. depilata* (from Gamboa, Panama), respectively **C, D** retrolateral tibia apophysis of *P. boliviensis* and *P. depilata*, respectively. Scales bars: 0.10 mm (**A**); 0.20 mm (**B**); 0.10 mm (**C**); 0.05 mm (**D**). C = conductor, E = embolus, IB = internal bulge, LL = locking lobes, MA = median apophysis, ST = subtegulum.

**Figure 10.**
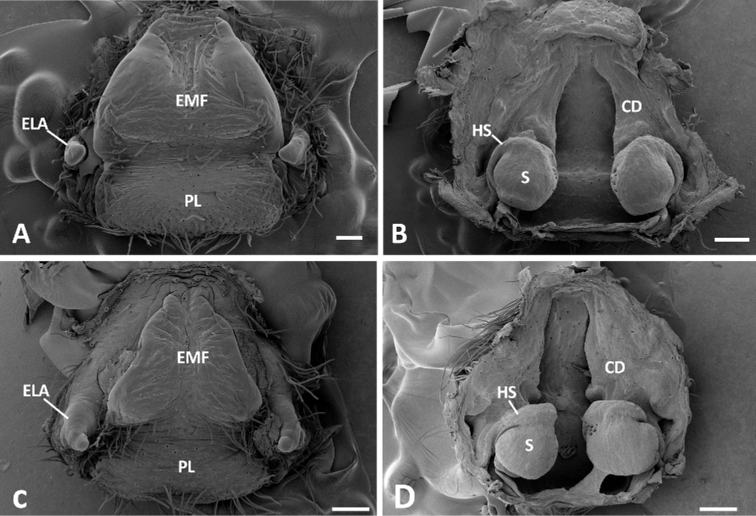
**A, B** epigynum and vulva (dorsal view) of *Phoneutria boliviensis* (from Pucallpa, Peru) **C, D** epigynum and vulva (dorsal view) of *Phoneutria depilata* (from Caimito, Esmeraldas, Ecuador). ELA = epigynal lateral apophysis, EMF = epigynal middle field, FD = fertilization duct, HS = head of spermatheca, PL = posterior lobe, S = spermatheca. Scales bars: 0.20 mm (**A**), 0.20 mm (**B**), 0.30 mm (**C**), 0.30 mm (**D**).

Figs 1A, B , 4C, D , 7A–C , 8A, B , 9B, D , 10C, D

Ctenus depilatus Strand, 1910. Holotype male from Colombia (ZMB 30615, examined). Valerio 1983: 101, fig. 2 (female).
Ctenus peregrinoides : Strand, 1910: 318 (syntypes: two females from Guatemala, in ZMB 30717, not examined). New synonymy.
Phoneutria depilata : Schmidt, 1954: 417-418.
Phoneutria colombiana Schmidt, 1956: 418; 1956: 28 (female holotype from Colombia, in SMF, not examined). New synonymy.
Phoneutria boliviensis : Simó & Brescovit, 2001: 74 (as senior synonym of P. depilata); Rozwałka, Rutkowski and Bielak-Bielecki 2017: 61, fig. 1b, c (female); Hazzi et al. 2018: 112, fig. 10D (male).
Phoneutria cf. boliviensis : Cathrine & Longhorn, 2017: 13, figs 1–6 (female).

###### Comments.

In their revision of *Phoneutria*Simó and Brescovit (2001) distinguished the Amazonian specimens of *Phoneutria boliviensis* from the specimens from Colombia and Central America (which we identify now as *P. depilata*) based on the epigynal morphology: “In specimens from Central America to Colombia it is triangular, with a wide base and a narrow apex, but in specimens from Ecuador to Bolivia the apex is more rounded”. Based on the fact that Simó & Brescovit were able to distinguish these epigynal morphological differences among these two *Phoneutria* species and that the only species of *Phoneutria* in the trans-Andean region is *P. depilata*, we suggest that *Ctenus peregrinoides* (from Guatemala) is a junior synonym of *P. depilata*. Strand described *Ctenus signativenter* in 1909 based on immature syntypes from Paramba, Ecuador (one male and two female syntypes, all immatures, 3500 ft, 28 April 1898, Rosenberg leg., in ZMB 306, not examined). We have designated *Ctenus signativenter* as a *nomen dubium* because the exact identity of this species cannot be ascertained with immature specimens, but we note that the type locality suggests that the *C. signativenter* syntypes belong to *P. depilata*. Based on the epigynal morphology (Schmidt 1956, fig.3), we synonymize *Phoneutria colombiana* with *P. depilata*. Both *peregrinoides* and *colombiana* had been synonymized with *P. boliviensis* by Simó and Brescovit (2001).

###### Other material examined.

**Nicaragua**: Región Autónoma de la Costa Caribe Sur: one female, Escondido River (12.1065°N, 84.0256°W, 10 m), 12.VII.1892, C.W. Richmond (USNM). **Panama**: Panama: one male, Pearls Island, San José (8.270219°N, -79.112038°W, 30 m), 02.IV.1944, J.P. Morrinson (USNM); Bocas del Toro: one female, Changinola, El Silencio (9.3845°N, 82.5356°W, 20 m), E. Marrango (USNM), one male and one female, Gamboa (9.1176°N. 79.6959°W, 50 m), 05.XIII.2018, N. Hazzi and S. Maneses (MCZ IZ 162179); Chiriquí: one male, one female, Puerto Amuelles (8.2841°N, 82.8691°W, 10 m), 25.VII.2018, N. Hazzi, J. Bernal, T. Rios (MCZ IZ 162180). **Costa Rica**: Alajuela: one male and one female, San Ramón, Muelle San Carlos (10.4335°N, 84.5622°W, 990 m) (MZUCR); one male, Canalete, Upala (10.8358°N, 85.0437°W, 950 m), 25.XI. 1979 (MZUCR), two females and two males, San Carlos, Peje Viejo (9.644°N, 82.7516°W), F. Garray (MZUCR), 00.X.1999; Limón: one female, Guapiles (10.2217°N, 83.7705°W, 450 m), 30.IX.1977 (MZUCR); one female, Batan (10.0842°N, 83.3364°W), 12.VIII.1984, Federico Muñoz (MZUCR), Reserva Biológica Hitoy Cerere (9.647°N, 83.0709°W); one female, Talamanca, Amumbri de Bratsi (9.6501°N, 82.7542°W) (MZUCR); Puntarenas: one female, Rincón de Osa (8.6986°N, 83.4876°W, 20 m), 00.III.1967, C. Valerio (MZUCR); one male, Conte, Casa de la Guardia Rural (8.443°N, 83.0401°W, 990 m), 14.VII.1984; one female and one male Cirenas (9.7199°N, 85.2119°W, 10 m), 00.VI.2018, N. Hazzi (MCZ IZ 162181), Cartago: one female, Turrialba, Bajo Pacuare (9.862°N, 83.5203°W, 730 m), 25.IV.1983, F. Calderón (MZUCR); Heredia: one juvenile, Sarapiqui, Reserva Tirimbina (10.4164°N, 84.1199°W, 160 m), 10.VI.2019, N. Hazzi; one male, San Isidro (10.0182°N, 84.0551°W, 1300 m) (MCZ IZ 162183). **Ecuador**: Esmeraldas: Esmeraldas, Caimito (0.7005°N, 80.0741°W, 10 m), 1.10.2019, N. Hazzi (MCZ IZ 162184). **Colombia**: Chocó: one female, Bahía Solano, Ciudad Mutis (6.2186°N, 77.4075°W, 5 m), 5.V.1973 (ICN-AR); one juvenile, Acandí, Capurgana (8.6338°N, 77.3503°W, 15 m), 08.X.2007, C. Duran (MPUJ); Cundinamarca: Fusagasugá (4.3439°N, 74.3678°W, 1600 m), 00.XII.2001 (ICN-AR-5258); Yacupi, vereda La Oscura (5.45°N, 74.35°W, 1190 m), 03.I.2000 (ICN-AR-907); Quipile, Vereda el Trigo (4.7455°N, 74.5341°W, 1300 m); 28.V.2000 (ICN-AR-908); Pandi, vereda El Caucho (4.1911°N, 74.4875°W, 910 m), 20.IX.2000 (ICN-AR-903); La Mesa (4.6333°N, 74.4666°W, 1080 m), 16.VI.1983 (ICN-AR-343); Nilo, Pueblo Nuevo (4.3166°N, 74.6333°W, 480 m), 12.I.1980 (ICN-AR-303); Santander: Chima, El Rodeo (6.3458°N, 73.3736°W, 113 m); 03.I.1970 (ICN-AR-315); one female, Suaita (6.10°N, 73.45°W, 1550 m), 10.V.1998 (ICN-AR-5261); Antioquia: one female, Urabá, Apartadó (7.8856°N, 76.6347°W, 20 m), 00.VII.2003 (ICN-AR-5259); one female, Urabá, Turbó (8.0981°N, 76.7317°W, 20 m) (ICN-AR-5260); one female, Urabá, Chigorodó (7.6769°N, 76.6864°W, 20 m), 00.IX.2003 (ICN-AR-5262); Cauca: one male, PNN. Gorgona Island (2.98°N, 78.1825°W, 5 m), 00.XII.2003 (ICN-AR-5263); one female and five males, Caloto, vereda Morales (3.0369°N, 76.4116°W, 1100 m), 00.X.2009, N. Muriel (MUSENUV); Valle del Cauca: one female and one male, Cali, El Aguacatal (3.4617°N, 76.5560°W, 1000 m), N.Hazzi (MUSENUV); one female, Cali (3.4616°N, 76.5560°W, 1000 m), 20.X.1982 (MUSENUV); one female, same locality 00.X.1986 (MUSENUV); one male, Cali, Barrio El Refugio (3.4372°N, 76.5225°W, 1000 m), 00.XI.1995(MUSENUV) one female, Dagua, El Palmar (3.6033°N, 76.6463°W, 1300 m), 27.IX.1994 (MUSENUV); one female, Roldanillo (4.4147°N, 76.1547°W, 950 m) (MUSENUV); KM 30, El Carmen (3.566°N, 76.6475°W, 1400 m); 10.XII.2008, N. Hazzi (MUSENUV); one male, Buga, Liceo de los Andes (3.8833°N, 76.2986°W, 950 m), 12.XII.2009, N. Hazzi (MUSENUV), one female, Buenaventura, Reserva Natural San Cipriano (3.8833°N, 76.9166°W, 180 m), 00.II.2012, N.Hazzi and J. Moreno (MUSENUV); Boyaca: Puerto Boyaca, Puerto Romero, vereda Los Quinchos (5.8375°N, 74.3408°W, 160 m); Risaralda: one female, Pereira (4.8133°N, 75.6961°W, 1400 m) (MUSENUV); one male, Balboa (4.9517°N, 75.9572°W, 1400 m); 10.XI.1998 (ICN-AR-5264); Quindío: one female, Montenegro, La Tebaida (4.5542°N, 75.7181°W, 1200 m), 27.I.2010, N. Hazzi (MUSENUV); one female, Montenegro, Pueblo Tapado (4.5178°N, 75.7847°W, 1250 m), 00.X.2004 (MUSENUV); Caldas: Samaná, Norcasia, Carrisa (5.5666°N, 74.8833°W, 600 m); 10.X.1992 (ICN-AR342).

###### Diagnosis.

Males of *P. depilata* resemble those of *P. boliviensis* by the truncated apex of the RTA (Fig. 9C, D), but differ from this and the remaining *Phoneutria* species by the lateral pronounced projection of the locking lobes visible in ventral view (Fig. 9B). In addition, males present an embolus with an internal bulge which is absent in *P. boliviensis*; and a much larger tegulum (Figs 7B, 9B). Females of *P. boliviensis* also resemble those of *P. depilata* by the general configuration of the epigynum but differ by the narrow area of the EMF (Fig. 8A), copulatory ducts slightly sclerotized (Fig. 8A), and large spermatheca heads (Figs 8B, 10D). In addition, both males and females of *P. depilata* can be distinguished from *P. boliviensis* and the remaining Amazonian species (*P. perty* and *P. fera*) by the four conspicuous series of yellow dots in the ventral side of the abdomen (this character is also present in *Phoneutria eickstedtae* Martins & Bertani, 2007).

###### Description.

**Male** (from Puerto Amuelles, Chiriquí, Panama, MCZ IZ 162180-1). Coloration (Figs 1B, 4C, D): Carapace brown with a longitudinal black line. Ocular area with brown setae and back oblique band from PLE to anterior dorsal shield of prosoma edge. Chelicerae brown with reddish setae. Sternum, endites and labium yellowish-brown. Abdomen yellow-brown dorsally, with yellow dots; ventrally dark brown with four conspicuous series of yellow dots. Total length 23.21. Carapace 12.38 long and 9.94 wide, eye diameters: AME 0.46, ALE 0.34, PME 0.55, PLE 0.55. Clypeal height 0.45, sternum 5.03 long, 4.65 wide; labium 1.62 long, 1.62 wide. Sternum 2.58 long and 2.50 wide, labium 1.99 long and 2.15 wide, endites 2.93 long and 1.70 wide. Leg measurements: I: femur 14.65, patella 5.77, tibia 15.75, metatarsus 14.31, tarsus 3.93, total 54.41; II: 13.72, 5.19, 13.85, 12.63, 3.30, total 48.69; III, 11.12, 4.95, 10.21, 10.09, 3.17, total 39.54; IV 13.56, 4.86, 13.25, 16.35, 3.96, total 51.98. Leg spination: I tibia v2-2-2-2-2, d1-1-1, p0-1-0, r1-1-0, metatarsus v2-2-2, p1-0-0 r1-0-0, II tibia v-2-2-2-2-2, d1-1-1, p1-1-0, r1-1-0, metatarsus v2-2-2, p1-0-0 r1-0-0, III v2-2-2, d1-1-1, p1-0-1-0, r1-0-1-0, metatarsus v2-2-2-2, p1-1-2, r1-1-2, IV tibia v2-2-2, d1-1-1, p1-0-1-0, r1-0-1-0, metatarsus v2-2-2-2, d0-1-0, p1-1-2, r1-1-2. Palp: RTA small and truncated at the apex (Figs 7C, 9D); embolus curve with internal bulge (Figs 7B, 9B); cup-shaped median apophysis constrain at the base (Figs 7B, 9B); conductor membranous, hyaline and C-shaped (Figs 7B, 9B); tegulum large with probasal rounded projection (Figs 7B, 9B).

**Female** (from Puerto Amuelles, Chiriquí, Panama, (MCZ IZ 162180-2). Coloration (Figs 1B, 4C, D): Carapace brown with a longitudinal black line. Ocular area with brown setae and back oblique band from PLE to anterior dorsal shield of prosoma edge. Chelicerae brown with reddish setae. Sternum, endites and labium yellowish-brown. Abdomen yellow-brown dorsally, with yellow dots; ventrally dark brown with four conspicuous series of yellow dots. Total length 25.77. Carapace 12.56 long and 9.82 wide, eye diameter: AME 0.47, ALE 0.36, PME 0.60, PLE 0.65. Clypeal height 0.89, sternum long 5.15 and 4.76 wide, endites 3.89 long and 2.30 wide, labium 1.33 long and 1.59 wide. Leg measurements: I: femur 13.43, patella 5.09, tibia 12.78, metatarsus 9.99, tarsus 3.33, total 44.62; II, 11.22, 5.00, 11.57, 9.30, 3.00, total 40.09; III 7.83, 4.00, 7.89, 7.60, 2.50, total 29.83; IV 12.00, 4.62, 9.83, 12.72, 3.15, total 42.32. Leg spination: tibia I–II v2-2-2-2-2, metatarsus I–II v2-2-2-2-2; III tibia v2-2-2, d1-1-1, p1-0-1-0, r1-0-1-0; metatarsus v2-2-2-2, p1-1-2, r1-1-2; IV tibia v2-2-2, d1-1-1, p1-0-1-0, r1-0-1-0, metatarsus v2-2-2-2, d0-1-0, p1-1-2, r1-1-2. Epigynum (Figs 6A, 10C): middle field convex with straight edges, anteriorly divergent and posteriorly convergent; lateral field with lateral apophysis. Vulva (Figs 6B, 10D): copulatory ducts slightly sclerotized, enlarged spermatheca heads, fertilization ducts small and posteriorly located.

**Figure 11.**
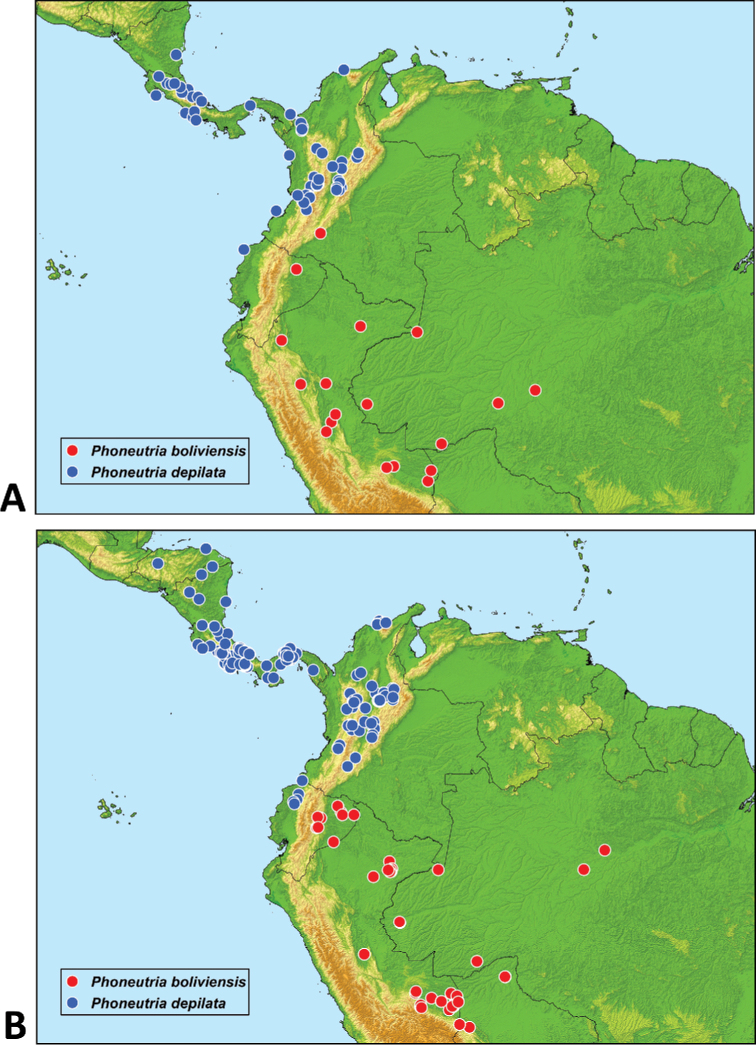
Occurrence records of *P. boliviensis* and *P. depilata* obtained from LIFIMU database (**A**) and iNaturalist (**B**).

###### Variation.

Males (n = 5): Total length 21.00–26.37, carapace 11.26–13.75, femur I 13.27–15.84. Females (n = 8): Total length 25.77–34.00, carapace 12.56–15.00, femur I 13.43–14.36.

###### Distribution.

Trans-Andean region (0–1700 m) in Ecuador, Colombia, Panama, Costa Rica, Nicaragua, Honduras and Guatemala.

###### Natural history.

This species is found in disturbed habitats associated with both dry and humid tropical forests (0–1700 m), usually on the ground with sparse litter and low vegetation (Hazzi 2014). The range of eggs per egg sac is 430–1300, and spiderlings emerge 28–34 days after the egg sacs are produced. Sexual maturity occurs after 14–17 molts, and spiders mature 300–465 days after emerging from the egg sac (Hazzi 2014). Valenzuela-Rojas et al. (2020) reported that *P. depilata* is an euryphagous predator with a broad diet made up predominantly of arthropods and to a lesser extent of small vertebrates (Gekkonidae, Hylidae, and Sphaerodactylidae). There are human bite records of this species reported in Costa Rica and in banana plantations in Colombia (Florez et al. 2003). All the cases reported have occurred with adults, and most of them have presented mild to moderate envenomation symptoms, with only one patient presenting severe symptoms such as renal failure (Florez et al. 2003). Estrada-Gomez et al. (2015) partially characterized the venom of this species, detecting Ctenitoxin-Pb48 and Ctenitoxin-Pb53, which showed a high homology with other Ctenitoxins (family Tx3) from *P. nigriventer*, *P. keyserlingi* and *P. reidyi* affecting voltage-gated calcium receptors (Cav 1, 2.1, 2.2 and 2.3) and NMDA-glutamate receptors. Valenzuela-Rojas et al. (2019) found that the venom of *P. depilata* was significantly more effective on vertebrate (geckos) than invertebrate (spiders) prey in both LD50 and immobilization time. In addition, males had slightly more toxic venom (LD50) to geckos and much more toxic venom to spiders when compared to females (Valenzuela-Rojas et al. 2019). For two periods, March to May and October to November, adult males and females with egg sacs are always found in homes in the Inter-Andean Cauca Valley of Colombia. This likely indicates two reproductive peaks that coincided with the two rainy seasons during those same periods (N. Hazzi, unpub. data).

**Figure 12.**
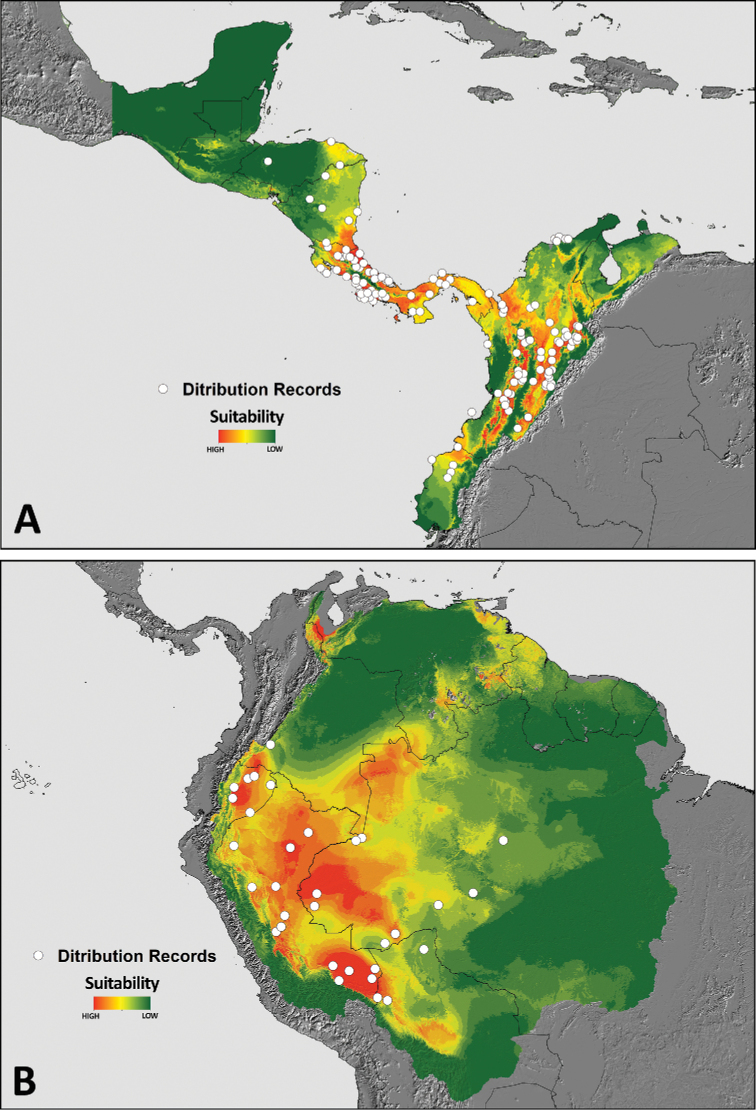
Distribution models **A, B** continuos model of *P. depilata* and *P. boliviensis*, respectively.

**Figure 13.**
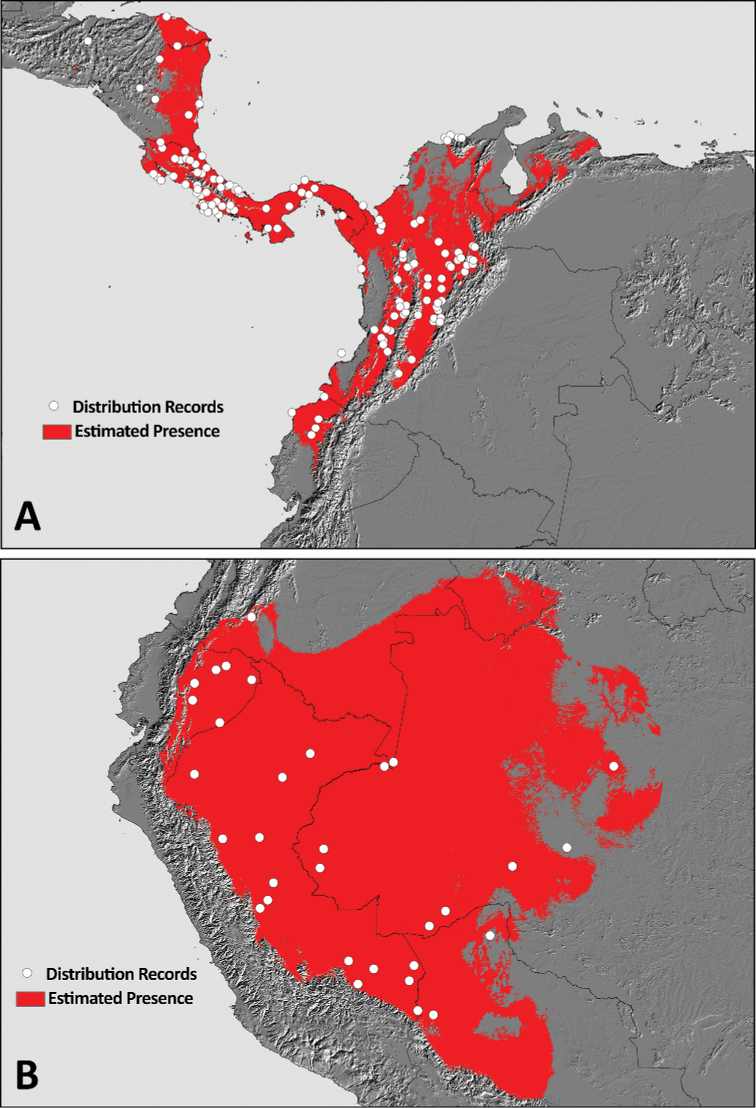
Distribution models **A, B** binary model (5% threshold) of *P. depilata* and *P. boliviensis*, respectively.

**Figure 14.**
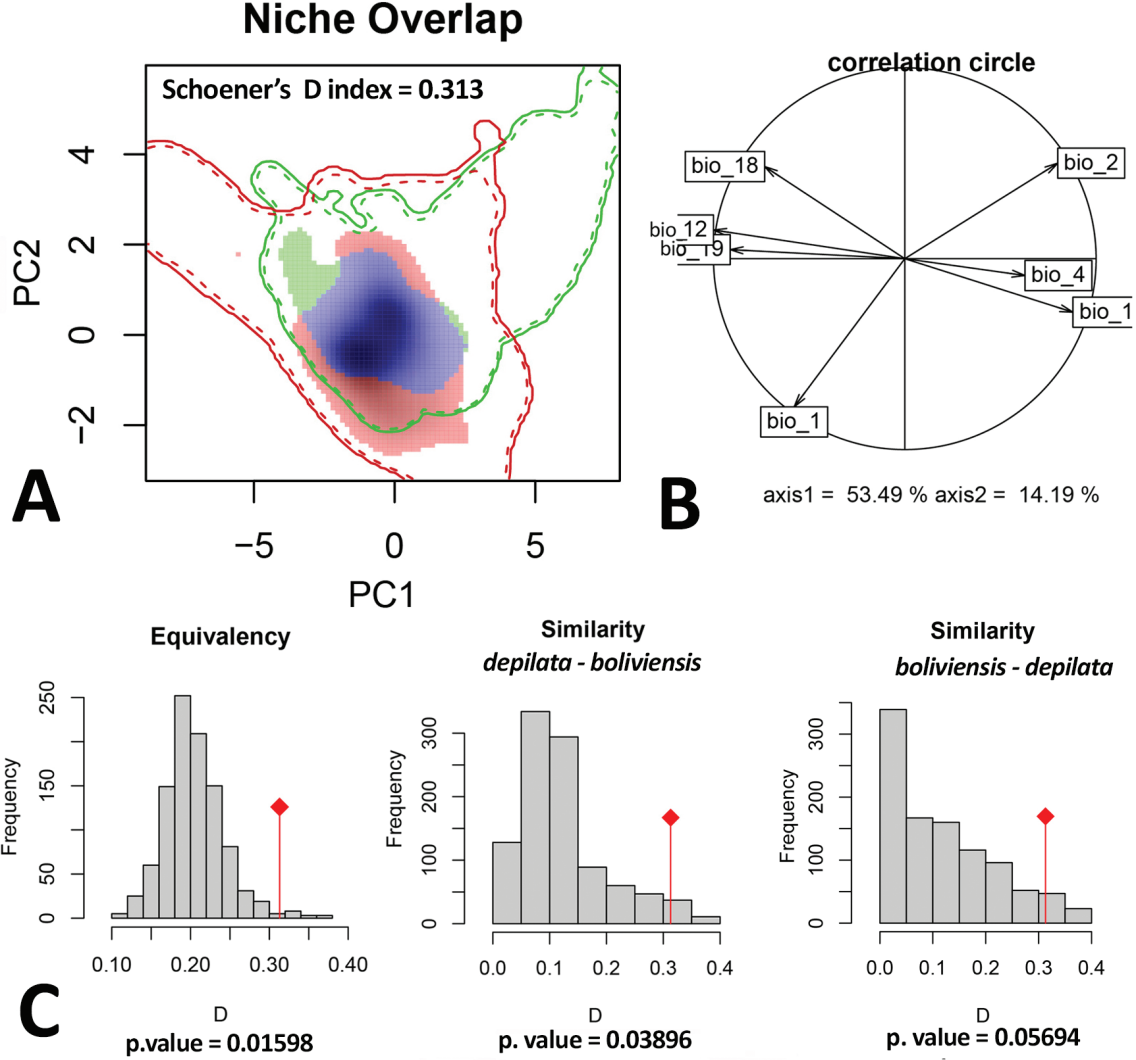
Equivalence and similarity tests in environmental space for *P. boliviensis* and *P. depilata***A** PCA of ecological climatic **B** the variables contribution to the analyses **C** graphical results of the equivalency and similarity permutation tests comparing the two species of *Phoneutria*. Line marks and filled squares are the available environment in each range (M) and occupied space by each species, respectively. Occupied climatic niche by *P. boliviensis*, *P. depilata* and niche overlap (D) are in green, red and blue colors, respectively.

## Discussion

Despite the medical importance of *Phoneutria*, its taxonomy and systematics have been always debated and there is still disagreement about the exact number of species in the genus. For instance, the last two taxonomic revisions of the genus contradict the boundaries of some species. Simó and Brescovit (2001) lumped several species into the medically relevant species *P. nigriventer* and only recognized five valid species. Martins and Bertani (2007) split *Phoneutria nigriventer* into three species, some of which had been recognized by other previous authors as valid. In the case of *Phoneutria depilata*, this species has been found co-occurring with *P. boliviensis* for several decades and many works on *P. depilata* have been published with the species misidentified as *P. boliviensis* (Valerio 1983; Hazzi et al. 2013; Hazzi 2014; Estrada-Gomez et al. 2015; Valenzuela-Rojas et al. 2019, 2020). The combination of detailed morphological (coloration and genitalia morphology) and molecular data has allowed us to distinguish *P. depilata* from *P. boliviensis*, and therefore reconsider the status *P. depilata* as a valid species.

Previous works of DNA barcoding in Lycosoidea have shown a range of genetic distances among congeneric species of 4–6.9% (Correa-Ramírez et al. 2010; Planas et al. 2013). Our analyses of the three species of *Phoneutria* resulted in interspecific distances between 6.1 to 8.2%, indicating similar genetic divergence to other Lycosoidea congeneric species. In addition, these divergences are also congruent with p-distances reported in other congeneric species of spiders (Barrett and Hebert 2005; Bidegaray-Batista et al. 2014; McHugh et al. 2014; Hormiga 2017; Agnarsson et al. 2018; Montes de Oca et al. 2016; Ballesteros and Hormiga 2018; Valdez-Mondragón 2020). Moreover, interspecific distances among haplotypes were, by far, higher than intraspecific variation between species haplotypes. For instance, the higher number of mutations was 6 between intraspecific haplotypes, compared with the lower number of mutations of interspecific species haplotypes (*P. boliviensis*-*P. fera* = 29). Interestingly, ITS-2 presented few segregating sites, and it was only able to differentiate haplotypes of *P. boliviensis* from the remaining two species just by one mutation step.

The distance-based method (ABGD) split *P. boliviensis* into two species, one which was not monophyletic. Several species delimitation studies in spiders have also shown that the ABGD method is sensitive to sampling and tends to over-split species when compared with other methods (Hamilton et al. 2014; Ortiz and Francke 2016; Valdez-Mondragón 2020). Instead, the phylogeny-based species delimitation methods employed in this study were congruent in identifying the three species of *Phoneutria*, corresponding completely with the morphological data. However, GMYC and mPTP methods grouped the three *Phoneutria* species into one, when only ITS-2 was used (an expected result due to the low genetic variation of this marker, as mentioned above). Because these two species delimitation methods were designed for single locus data (Pons et al. 2006; Fujisawa and Barraclough 2013; Kapli et al. 2017), we also implemented the BPP which explicitly models the evolution of multilocus data (Yang 2015; Luo et al. 2018). The results of this analysis also supported the existence of three species of *Phoneutria*. Although the ITS-2 has rarely been used in studies of spiders compared to other nDNA markers (e.g. 28S and histone H3), several studies have started to use it for DNA barcode and species delimitation recently. In *Anelosimus* species (Agnarsson 2010) and *Gasteracantha cancriformis* (Chamberland et al. 2020), this marker has insufficient variation to resolve relationships within species and among closely related species. However, for species of the genus *Theridion*, ITS-2 has shown a perfect match with the morphology-based species delimitation (Domènech et al. 2020). In addition, this marker has also shown to be informative with species of *Loxosceles* (Valdez-Mondragón et al. 2019). Therefore, ITS-2 sometimes can be useful for species identification and delimitation, and it should be used together with COI.

Citizen science platforms have provided unprecedented access to documenting species diversity and distribution across the world (Amano et al. 2016). In the case of iNaturalist, this platform presents more than 46,765,000 observations of more than 291,200 putative species of animals and plants (Horn et al. 2018). Recently, various studies have used this platform to detect disease in red mangroves (Rossi 2017), document biodiversity and distribution of echinoderms and termites (Michonneau and Paulay 2015; Hochmair et al. 2020), the rediscovery of threatened rare species (Wilson et al. 2020), and the discovery and description of new species (Winterton 2020). To our knowledge, this is the first study that has used iNaturalist to gather occurrence records on venomous species to estimate distribution models. For the two species of *Phoneutria* studied here, iNaturalist presented higher and more widely distributed records than our database, compiled using literature, examination of specimens from different museums, and years of personal fieldwork. Thus, our study demonstrated iNaturalist’s ability to gather occurrence records and improve distribution knowledge of conspicuous and large, venomous spiders that inhabit synanthropic environments, like species of *Phoneutria*. Unfortunately, for the two remaining Amazonian species of *Phoneuria* (*P. reidyi* and *P. fera*), based on our limited knowledge, it is only possible to distinguish these two species with genitalia images and not with photographs of the habitus at this time. Therefore, we were not able to include the information of iNaturalist to model their potential distribution.

*Phoneutria boliviensis* and *P. depilata* live in lowland areas, and sometimes premontane ecosystems as well (Valerio 1983; Simó and Brescovit 2001; Hazzi et al. 2013). The distribution models corroborate that suitable areas for both species are lowland rainforest ecosystems. However, the model also indicated dry and premontane tropical ecosystems reaching elevations of 1600 m as suitable areas for *P. depilata*, which is congruent with the occurrence records and previous observations about the wide niche plasticity of this species (Hazzi 2014). It is also important to highlight that the species distribution maps (SDM) indicated that a large area of the Pacific of Colombia is unsuitable for this species. However, we think that the species may be present along this area but there are no records as this is one of the less explored regions of this country (Arbeláez-Cortés 2013; Arbeláez-Cortés et al. 2017). The compiled occurrence records and SDMs obtained for these two species, together with the morphological diagnosis, could have significant use in identifying risk areas of accidental bites and help health care personnel determine the species involved, especially for *P. depilata* which has been involved in bite accidents (Trejos et al. 1971; Florez et al. 2003).

Phylogenetic niche conservatism has been suggested as one of the potential forces in speciation and species richness patterns in the tropics (e.g., Wiens and Graham 2005; Wiens et al. 2011; Pyron et al. 2015). Under the allopatric speciation model, especially when allopatric lowland taxa are separated by a geographic barriers, one may expect that the tendency of species to maintain their ancestral climatic niche prevents them from adapting to new environments (such as mountains), isolating, and promoting speciation. (Wiens 2004; Pyron et al. 2015; Posso-Terranova and Andrés 2016). *Phoneutria depilata* has an allopatric distribution with respect to the three Amazonian species of *Phoneutria* (*P. fera*, *P. boliviensis* and *P. reidyi*). The Andes works as the geographic barrier that separates *P. depilata* (trans-Andean species) from the Amazonian species (cis-Andean species), a biogeographic pattern commonly see with many other Neotropical taxa (Albert et al. 2006; Weir and Price 2011; Richardson et al. 2015; Bartoleti et al. 2018; Salgado-Roa et al. 2018). The niche comparison analysis of these two species, using equivalency and similarity tests, indicated that both species presented niche conservatism. However, the phylogenetic analyses using different optimality criteria were not able to support, with high confidence, that *P. depilata* and *P. boliviensis* are sister species. In addition, we did not have samples of *P. reidyi*. Nevertheless, we think that it is still possible to conclude that Amazonian and the trans-Andean species of *Phoneutria* have conserved their climatic niches because the three Amazonian species are sympatric, occupying the same kind of ecosystems (climatic areas). Furthermore, the allopatric species *P. depilata* has a climatic niche similar to the Amazonian species *P. boliviensis*. These results are also congruent with other allopatric lowland cis and trans-Andean taxa that have conserved their climatic niches (Albert 2010; Richardson et al. 2015).

In conclusion, using morphological and molecular data, together with species delimitation methods our study revalidates *Phoneutria depilata* as a valid species separate from *P. boliviensis*. Both species have allopatric distributions separated by the Andean mountains, and species distribution models indicated lowland tropical rain forest ecosystems as the most suitable environments for these species. In addition, this work demonstrated the value of citizen science platforms like iNaturalist for occurrence records and improving species distribution knowledge. *Phoneutria depilata* and the three Amazonian species presented niche conservatism following the expected neutral model of allopatric speciation. Finally, the morphological diagnosis of these two species and the distribution maps provided in this work will be useful for future studies in venom, epidemiology of bites, and systematics of this venomous groups of spiders.

## Supplementary Material

XML Treatment for Phoneutria

XML Treatment for Phoneutria boliviensis

XML Treatment for Phoneutria depilata

## Supplementary materials

Supplementary material 1 Concatenated alignment Data type DNA alignment Explanation note Concatenated DNA Alignment of ITS-2 and COI. https://binary.pensoft.net/file/517670This dataset is made available under the Open Database License (http://opendatacommons.org/licenses/odbl/1.0/). The Open Database License (ODbL) is a license agreement intended to allow users to freely share, modify, and use this Dataset while maintaining this same freedom for others, provided that the original source and author(s) are credited.Nicolas A. Hazzi, Gustavo Hormiga

Supplementary material 2 Curated iNaturalist database for *Phoneutria boliviensis* and *P. depilata* Data type Occurences Explanation note Curated iNaturalist database for *Phoneutria boliviensis* and *P. depilata*. Accessed in July 2020. https://binary.pensoft.net/file/517671This dataset is made available under the Open Database License (http://opendatacommons.org/licenses/odbl/1.0/). The Open Database License (ODbL) is a license agreement intended to allow users to freely share, modify, and use this Dataset while maintaining this same freedom for others, provided that the original source and author(s) are credited.Nicolas A. Hazzi, Gustavo Hormiga
