# Corrigenda: Morphological and molecular evidence support the taxonomic separation of the medically important Neotropical spiders *Phoneutria depilata* (Strand, 1909) and *P. boliviensis* (F.O. Pickard-Cambridge, 1897) (Araneae, Ctenidae). ZooKeys 1022: 13–50. https://doi.org/10.3897/zookeys.1022.60571

**DOI:** 10.3897/zookeys.1033.65850

**Published:** 2021-04-22

**Authors:** Nicolas A. Hazzi, Gustavo Hormiga

**Affiliations:** 1 The George Washington University, Department of Biological Sciences, Washington, D.C. 20052, USA The George Washington University Washington DC United States of America; 2 Fundación Ecotonos, Cra 72 No. 13ª-56, Cali, Colombia Fundación Ecotonos Cali Colombia

## Abstract

In a recent publication (Hazzi & Hormiga 2021) we demonstrated that there are two species under the name of the ctenid spider *Phoneutria boliviensis* (F.O. Pickard-Cambridge, 1897) and used the name *Phoneutria depilata* (Strand, 1909) for this second species, which in the past had been treated as a junior synonym of *boliviensis*. We proposed that “*Ctenus peregrinoides* Strand, 1910*”* was a new junior synonym of *Phoneutria depilata*. We note here that such synonymy is an error. Strand did not use the name “*Ctenus peregrinoides*” as valid when he proposed it (valid as opposed to, for example, “if this were a new species, here is a name”), which means that his provisional name is not available for nomenclatural purposes. Thus, formally treating “*peregrinoides”* as a junior synonym is inconsequential because Strand’s name is not available in a nomenclatural sense

In a recent publication (Hazzi and Hormiga 2021) we demonstrated that there are two species under the name of the ctenid spider *Phoneutria boliviensis* (F.O. Pickard-Cambridge, 1897) and used the name *Phoneutria depilata* (Strand, 1909) for this second species, which in the past had been treated as a junior synonym of *boliviensis*. We proposed that “*Ctenus peregrinoides* Strand, 1910” was a new junior synonym of *Phoneutria depilata*. We note here that such synonymy is an error. The problem resides in the fact that the name “*Ctenus peregrinoides*” is not available based on the Criteria of Availability of the ICZN (1999), of which Articles 10 and 11–20 must be satisfied. Article 11.5 states that: “Names to be used as valid when proposed. To be available, a name must be used as valid for a taxon when proposed, unless it was first published as a junior synonym and subsequently made available under the provisions of Article 11.6.1” (ICZN 1999). This requirement is not satisfied by Strand’s provisional name “*C. peregrinoides*.” Strand (1909a: 318) described two females from Guatemala under the name *Ctenus peregrinus* F.O. Pickard-Cambridge, 1900, and noted some differences with Pickard-Cambridge’s illustration of *peregrinus* in ‘Biologia Centrali Americana’, stating that “Sollte die Art neu sein, möge sie *peregrinoides* m. genannt warden” (“If the species is new, it may be called *peregrinoides*”). Thus, Strand did not use the name “*Ctenus peregrinoides*” as valid when he proposed it (valid as opposed to, for example, “if this were a new species, here is a name”), which means that his provisional name is not available for nomenclatural purposes. Formally treating “*peregrinoides*” as a junior synonym is inconsequential because Strand’s name is not available in a nomenclatural sense.

Furthermore, we need to correct the exact publication dates of the two relevant works of Strand (1909a, b). The unavailable name “*peregrinoides*” was published on 21 October,1909. A couple of months later (21 December 1909) Strand described *Ctenus depilatus*. The fact that these two publications appeared both in the 28^th^ issue of Zoologische Jahrbücher, Abteilung für Systematik, Geographie und Biologie der Tiere, which corresponded to 1910 (as printed in the frontispiece of the journal), has been a source of confusion regarding the publication dates. While Roewer (1955: 649 for *C. depilatus*; 654 for *C. peregrinus*) correctly lists both of them as published in 1909, the catalogs of Petrunkevitch (1911: 413, 476) and Bonnet (1956: 1279, 1287) list them as published in 1910.
